# Public transport use cessation and age-related functional decline: a quasi-causal analysis using the English longitudinal study of ageing

**DOI:** 10.1186/s12889-026-27249-9

**Published:** 2026-04-25

**Authors:** Omer Dilian, Paul D. Feigin, Nadav Davidovitch, Karel Martens

**Affiliations:** 1 Faculty of Architecture and Town Planning, Technion – Israel Institute of Technology, Haifa, Israel; 2 Faculty of Data and Decision Sciences, Technion – Israel Institute of Technology, Haifa, Israel; 3 Faculty of Medicine, Bar Ilan University, Safed, Israel

**Keywords:** Public transport, Transport and health, Public policy, Older adults, Mobility, Gait speed, Cognitive function, Natural experiment

## Abstract

**Background:**

Mobility is crucial for older adults’ independence, health and wellbeing, and depends on access to suitable transport. As people age, they often stop using public transport, yet this cessation’s health consequences are poorly understood. A key challenge is bidirectionality: declining health both cause and result from cessation. To address this, a quasi-experimental approach was utilised, focussing on cessation caused by structural, non-health-related factors, aiming to quantify impacts on two major markers of ageing: gait speed and cognitive function.

**Methods:**

Participants from waves 3–10 of the English Longitudinal Study of Ageing (ELSA) were classified according to their public transport use status, with ceasers further classified by cessation reason. Linear mixed-effects models were used to examine associations between public transport use cessation and health outcomes, testing for changes in the rate of decline following cessation, adjusting for age, sex, and relevant covariates.

**Results:**

Out of 18,292 unique participants 3,400 ceased using public transport, 794 of them for structural reasons. Structural cessation was significantly associated with accelerated gait speed and cognitive declines (β=-0.001 m/s /year, *p* = 0.008; β=-0.012/year, *p* = 0.002 respectively), compared to pre-cessation. In contrast, health-related ceasers exhibited sharp declines at cessation, with insignificant subsequent changes in rate.

**Conclusions:**

Public transport use cessation is a relatively common phenomenon, affecting almost a fifth of participants. While often caused by health-related reasons, cessation also independently leads to adverse health outcomes, specifically accelerated physical and cognitive declines. Policy for maintaining accessible and adequate public transport is essential for preserving mobility and might delay these declines.

## Introduction

Mobility is one of the most important capacities related to healthy ageing, and age-related mobility decline has been linked to numerous adverse health outcomes in older adults, including mortality, morbidity, disability, and reduced quality of life [1–4]. Meaningful mobility, as in the ability to reach places one is interested in reaching, is usually dependent on the ability to use different transport modes: driving, using public transport, or cycling, among others [4, 5]. Yet, this ability significantly declines with ageing, due to several well-known physiological, pathological and social processes: physical, cognitive, sensory and functional declines [4, 6, 7].

While the importance of losing the ability to use a mode of transport, and its impacts on health and wellbeing, are well recognised, research in this field has been mainly focussed on driving. Driving cessation, the process of giving up driving, has been linked to multiple adverse health outcomes, including accelerated cognitive decline, increased social isolation and increased morbidity and mortality [8–11]. However, insight into use cessation of other modes of transport remains generally scarce, with a small number of studies qualitatively examining cycling and public transport use cessation [12, 13].

Public transport is of special importance to older adults’ mobility, as in addition to the share of the population that does not have access to a car, it allows older adults that ceased driving to retain motorised mobility further into the ageing process [14–16]. Additionally, as opposed to more active modes of transport, such as cycling, it sometimes allows more frail older adults to continue moving relatively independently and over larger distances, even after significant functional decline. However, quantifying the impacts of public transport use on older adults’ health has proven difficult. The relationship between public transport use and health is highly bidirectional; that is, while public transport use might lead to better health outcomes via mechanisms such as increased physical activity, trip making, involvement in out-of-home activities and social participation and reduced social isolation [17–19], it is just as likely that better health leads to increased public transport use – as health is correlated with overall increased trip making and social participation [20], and it is easier for healthier persons to walk, board, and access public transport infrastructure and services [21]. This bidirectionality makes it difficult to distinguish between the causality of interest (public transport use or cessation and their health impacts) and the reverse causality (health impacts on public transport use). In several studies examining these relationships, older adults’ public transport use has been associated with health benefits such as reduced obesity and depression and increased gait speed and cognitive function (for a comprehensive systematic review, see [17]). Yet, many of these studies utilised cross-sectional designs, unfit to distinguish between the two directions of causality.

In a recent study by the authors of the current paper, public transport use cessation was examined qualitatively, with findings underlining that cessation has potential detrimental effects on older adults’ mobility and wellbeing [12]. Due to its qualitative nature, cessation’s actual effects on health could not be assessed. Thus, the aim of this study is to quantify public transport use cessation’s effects on two prominent markers of functional ageing linked with public transport use: cognitive function and gait speed. To tackle the issue of bidirectionality, this study employs a longitudinal approach, discerning between two groups of public transport use ceasers: (1) those that ceased using public transport due to existing health issues, and (2) those that ceased due to unrelated, ‘structural’ reasons, such as no longer having available public transport. These ‘structural ceasers’ are then employed as an instrumental quasi-experimental group, with the assumption that within them, health was not a reason to cessation, and health changes following cessation can be understood, at least partly, as a result of cessation and not a cause for it [22]. This study’s main hypothesis, thus, is that older adults who ceased using public transport due to *structural* reasons will experience accelerated gait speed and cognitive function declines, relative to the average age-related decline trajectory.

## Methods

### Data

The data used in this study comes from waves 3–10 of the English Longitudinal Study of Ageing (ELSA) [23]. ELSA contains data of a nationally representative sample of adults aged 50 or more, living in private households in England. Participants are interviewed and undergo an extensive set of physical and biological measurements biennially. Data collection for wave 3, the first to be included in this study, began in 2006, and data collection for wave 10, the last to be included in this study, ended in 2023.

Data from waves 1 and 2 were omitted to minimise risk of bias, as the question regarding public transport use frequency, central to the analysis conducted in this paper, was coded differently in these first waves. The data used in all waves was collected through face-to-face interviews with participants, in addition to self-completion questionnaires. Detailed documentation, and access to the ELSA database, are available on the ELSA website (https://www.elsa-project.ac.uk/).

### Variables

*Gait Speed*, in metres/second, was measured among all participants aged 60 or older in all waves of ELSA. Participants were requested to walk a 2.4 m track at their usual pace, with the interviewer timing their walk. This was conducted twice for each participant in each wave, and the variable used in this study is a calculated mean of these two assessments.

*Cognitive Function* is measured in ELSA using an array of variables corresponding to various cognitive capabilities, including executive function, memory, processing speed, visual accuracy, and more. However, not all tests were conducted consistently in all waves. Thus, and following previous studies using ELSA’s cognitive function data [24, 25], we created a composite score based on participants’ score in the word recall test (memory) and the animal naming test (executive function), available in all waves used in this study.

In the word recall test, participants are presented with 10 words, and are required to recall them both immediately, and after a short pause, during which they continue to undergo the cognitive tests battery. Participants are scored between 0 and 10 according to the number of words they successfully recalled, in both the immediate and the delayed test. In the animal naming test, participants are asked to name as many animals as they can in a one-minute period, with each different animal name worth 1 point in their final score. The three tests were individually transformed into z-scores, standardized, and an average was calculated to represent overall cognitive function, with higher scores representing better function.

### Public transport use and cessation

Public transport use frequency is routinely asked in every ELSA wave, using the same question since wave 3: “How often do you use public transport”, with answers arranged in six levels from “every day” to “never”. In this study, public transport use cessation was defined as the point in time in which a participant who, up until this point, had used public transport, reported no longer using it, without returning to use it in a later wave. To operationalise this definition, participants were assigned a binary public transport use variable in all their available waves (user or non-user). In each given wave, a participant was considered a public transport user if they had answered the frequency question with any frequency *higher* than “once a month or less”. Participants that in a certain wave answered they used public transport “once a month or less” were considered non-users for that wave, as such a frequency represents very little actual public transport use, and was deemed closer to non-use than to use.

For each participant, the individual binary variables were arrayed to create a sequence of public transport use along available waves. Then, each participant was assigned a use status: always-users, for participants who reported using public transport along all available waves; never-users, for participants who reported not using public transport along all available waves; adopters, for participants who reported not using public transport up until a certain wave, then started using it afterwards; and ceasers, for participants who reported using public transport up until a certain wave, and not using it afterwards. Ceasers were also assigned a cessation wave – the first wave where they reported not using public transport anymore.

In each ELSA wave, participants who reported a very low frequency of public transport use (“never” or “once a month or less”) were further asked why they did not use public transport. Participants were given a list of 12 potential reasons for not using public transport, with free-speech answers later coded to one of the 12 reasons by the interviewer. Following [19], the following reasons were considered of structural nature: no available public transport; public transport does not go where needed; public transport is too expensive; public transport is too unreliable; public transport is too infrequent; fear of crime in public transport; and public transport is too dirty. The following cessation reasons were considered to be of health-related nature: participant’s health prevents them from using public transport; and participant did not use public transport due to mobility problems. Other reasons available in ELSA, not falling into these two categories, were: participant prefers walking; participant does not need to; and it is too inconvenient. While the last could be thought of as structural, it was excluded in order to minimise health-related confounding, as in some cases it could mask health-related reasons such as declines in physical or functional capabilities, that led to inconvenience. Participants who ceased using public transport for one of these reasons were excluded from the analyses in this study. Participants were able to give multiple answers for this question. Thus, participants who gave both a structural and a health-related reason were excluded from analyses.

### Covariates

Covariates included: age; sex; access to car (either as a passenger or as a driver); total number of persons living in the household; presence of any long-term illness by self-report; total non-pension wealth; presence of one or more listed cardiovascular diseases (high blood pressure, angina, heart attack, congestive heart failure, heart murmur, abnormal heart rhythm, stroke, high cholesterol); presence of one or more listed non-communicable diseases (lung diseases, asthma, arthritis, osteoporosis, cancer, Parkinson’s disease, psychiatric diseases, Alzheimer’s disease, or other dementias); difficulty in one or more activities of daily living (ADLs); and difficulty in one or more intermediate activities of daily living (IADLs).

### Statistical analysis

Since walking speed and cognitive function vary substantially both between individuals and within individuals over time, a mixed-effects model approach was chosen. Models included person-level random intercepts to account for the non-independence of repeated measures and the fact that each subject may have a different starting gait or cognitive ability. The parameters of the mixed model regression were estimated by maximum likelihood. Each outcome was regressed on chronological age, a binary indicator for cessation and a term representing time since cessation, alongside the above-described covariates. Separate models were fitted for cessation due to structural and health-related reasons. In these models, always-users were analysed jointly with ceasers. For gait speed analyses, data was used from participants aged 60 and higher, due to gait speed being measured only from this age. For cognitive function analyses, data from all participants, regardless of age, was used. Models were fitted using the lme4 package in R version 4.5.1.

To illustrate counterfactual trajectories, age- and sex-specific covariate trends were derived from the always-user group. Using these trends, prediction plots were then produced from the fitted models to depict predicted declines in health outcomes for cessation in selected ages (60, 70 and 80), and plotted against the always-user reference.

## Results

### Sample characteristics

Overall, the cleaned database contained data from 18,292 unique participants over 76,132 observations. Of these, 20,875 observations of 3,400 unique participants belonged to ceasers, and 11,902 observations of 3,545 unique participants belonged to always-users. This represents a prevalence of 18.6% for public transport use cessation. Data belonging to adopters and never-users was not used in this study. As elaborated in the previous section, ceasers were divided into subgroups according to their reason for cessation, with 648 ceasers ceasing due to health- related reasons, and 794 due to structural reasons. Ceasers who ceased using public transport because of another reason available in the ELSA database (such as “no need for public transport”) were not used in this study.

Table 1 describes the cohort’s characteristics, divided to the three main examined subgroups – always-users, structural ceasers, and health-related ceasers. Global ANOVA and χ² test indicated that all variables differed significantly between the groups (*p* < 0.0001), as was expected considering group definition. It can be seen that in general, structural ceasers and always-users are relatively similar in age (65.6 ± 10.4 vs. 66.4 ± 11.4 years) and gender distribution (54.7% vs. 59.5% female), with structural ceasers having slightly better gait speed (0.923 ± 0.266 vs. 0.84 ± 0.262 m/s) and cognitive function (0.326 ± 0.672 vs. 0.05 ± 0.72). Two covariates stand out as markedly different between structural ceasers and always-users – wealth quintiles, with structural ceasers generally wealthier than always-users; and car availability, with 93.7% of structural ceasers reporting having a car available for their use, compared with 65.9% for always users. These suggest that structural ceasers are, on average, a relatively well-off group, and could mean that choice was involved in their decision to stop using public transport (owing to having other options). Other covariates were broadly comparable between structural ceasers and always-users.

**Table 1:** Cohort characteristics

	Ceasers (structural)	Ceasers (health-related)	Always-users
Unique participants	794	648	3545
Number of observations	5127	3683	11,902
Median number of observations for participant	7	6	2

	Average ± SD / %	Average (± SD) / %	Average (± SD) / %
Age	65.6 ± 10.4	69.7 ± 20.4	66.4 ± 11.4
Sex (female)	54.7%	70.2%	59.5%
Car availability	93.7%	56.4%	65.9%
Presence of long-standing illness	50.1%	80.5%	52.6%
Wealth quintile			
1	9.4%	34.8%	23.3%
2	12.5%	23.9%	21.9%
3	19.5%	19.2%	19.8%
4	27.3%	13.9%	17.9%
5	31.3%	8.2%	17.2%
CVD Presence	43.4%	67.8%	48.1%
NCD Presence	43.6%	73.7%	46.2%
Total number of people living in household	2.1 ± 0.9	1.6 ± 0.9	2 ± 0.9
Difficulty in 1 or more ADL	11.6%	46.8%	14.8%
Difficulty in 1 or more IADL	12.2%	54.9%	16.1%
Gait speed (m/s)	0.923 ± 0.266	0.622 ± 0.245	0.84 ± 0.262
Cognitive function	0.326 ± 0.672	-0.317 ± 0.722	0.05 ± 0.72

The values represent aggregated results from all observations. For Gait speed, values are for participants aged 60+

*CVD* Cardiovascular disease, *NCD* Non-communicable disease, *IADL* Intermediate activities of daily living, *ADL* Activities of daily living

In contrast, health-related ceasers were, on average, older (69.7 ± 20.4 years), more likely to be female (70.2%), and had significantly worse health scores: a mean gait speed of 0.622 ± 0.245 m/s and a mean cognitive function score of -0.317 ± 0.722. Health-ceasers also had a higher prevalence of comorbidities, difficulties in ADLs and IADLs, and lower wealth, reflecting an overall poorer health profile relative to the other groups.

### Public transport use cessation and health outcomes

Tables 2 and 3 report the results of the linear mixed effects models for gait speed and cognitive function, respectively. Age was significantly linked to lower gait speed and cognitive function, consistent with established age-related health declines. Multiple covariates were also found significantly related to the analysed health outcomes; in the gait speed model, being female, living in a large household, reporting having a long-standing illness or difficulties in ADLs and IADLs, were all linked with lower gait speed. Having access to a car and being in higher wealth quintiles were both linked with slower declines. In the cognitive function model, only age and reported difficulties in ADLs and IADLs were significantly and negatively linked with cognitive function; while being female, having access to a car and being in higher wealth quintiles were linked with higher function.

**Table 2:** Linear mixed-effects model for gait speed

Fixed Effects	Estimate	Standard Error	*p* value
(Intercept)	1.68	0.038	< 0.001***
Age	**-0.012**	**0.0004**	< 0.001***
After Cessation (binary)	0.001	0.009	0.891
Time since cessation	**-0.001**	**0.0004**	0.008**
Sex	**-0.04**	**0.008**	< 0.001***
Car availability	**0.044**	**0.007**	**< 0.001**
Total number of people living in household	**-0.011**	**0.004**	0.008**
Long standing illness presence	**-0.025**	**0.005**	< 0.001***
Wealth quintile	**0.029**	**0.003**	< 0.001***
CVD presence	0.002	0.005	0.743
NCD presence	0.007	0.006	0.258
Difficulty in IADL	**-0.07**	**0.007**	< 0.001***
Difficulty in ADL	**-0.061**	**0.007**	< 0.001***

Significant variables are marked bold. Gait model based on 8,373 observations of 2,118 unique participants, age ≥ 60

*CVD* Cardiovascular disease, *NCD* Non-communicable disease, *IADL* Intermediate activities of daily living, *ADL* Activities of daily living

^*^p < 0.05, ^**^p < 0.01, ^***^p < 0.001

**Table 3:** Linear mixed-effects model for cognitive function

Fixed Effect	Estimate	Standard Error	*p* value
(Intercept)	-0.019	0.064	0.768
Age	**-0.006**	**0.001**	< 0.001 ***
After Cessation (binary)	-0.023	0.023	0.312
Time since cessation	**-0.012**	**0.004**	0.002 **
Sex	**0.103**	**0.024**	< 0.001 ***
Car availability	**0.183**	**0.017**	< 0.001 ***
Total number of people living in household	0.007	0.009	0.419
Long standing illness presence	-0.002	0.012	0.897
Wealth quintile	**0.077**	**0.006**	< 0.001 ***
CVD presence	-0.012	0.012	0.324
NCD presence	0.023	0.013	0.065
Difficulty in IADL	**-0.120**	**0.016**	< 0.001 ***
Difficulty in ADL	**-0.050**	**0.017**	0.003 **

Significant variables are marked bold. Cognitive function model based on 11,214 observations of 2,506 unique participants, age ≥ 50

*CVD* Cardiovascular disease, *NCD* Non-communicable disease, *IADL* Intermediate activities of daily living, *ADL* Activities of daily living

^*^p < 0.05, ^**^p < 0.01, ^***^p < 0.001

With respect to the link between cessation and gait speed and cognitive function, the binary term for being after cessation, inserted to potentially reflect an immediate drop in health after cessation was found to be insignificant, for both outcomes, among structural ceasers. However, time since cessation was significantly and negatively associated with both gait speed and cognitive function – representing a sharper decline of 0.001 m/s and 0.012 points on the cognitive function score, per year, following cessation.

**Fig. 1:**
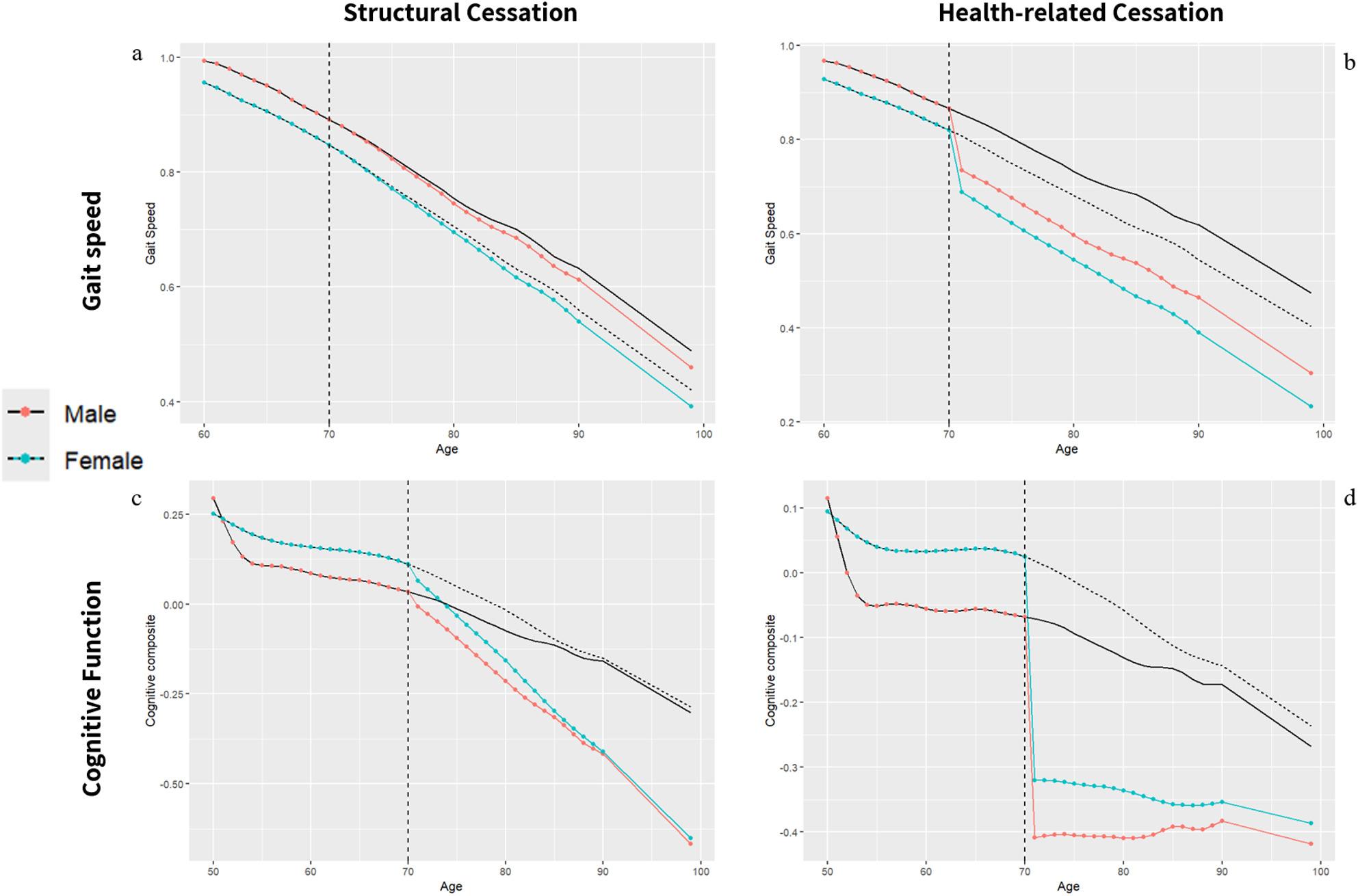
Predicted trajectories of health outcomes by age and sex for structural and health-related public transport use cessation, cessation age set at 70. Black lines represent projected trajectories for always-users; with dotted lines for females and continuous lines for males. Colored lines with crosses represent trajectories for ceasers; blue for females and red for males

Figure 1 (a-d) represents the predicted trajectories of health outcomes by age and sex, for structural and health-related public transport use cessation. For these plots, baseline population profiles of covariate trends by age and sex were created, based on data from the always-user group, reflecting change trends in the general user population unrelated to any type of cessation. These were used to predict the effects of cessation on declines, simulating cessation at a certain age, using the above models. Figure 1a and c depict the effects of structural cessation on gait speed and cognitive function, respectively. After cessation, the ceasers’ trajectory quite clearly diverges from the always-users baseline, indicating accelerated gait speed and cognitive function declines. This effect, as seen in the above models, is more pronounced for cognitive function.

Figure 1b and d show the same predictions for health-related ceasers, and indicate a very different pattern; among health-related ceasers, health outcomes decline sharply simultaneously with cessation, with smaller changes in the decline slope thereafter. This sharp decline likely represents the event that has caused these participants to cease using public transport, rather than an effect of cessation on the outcome; further interpretation is provided in the discussion section of this manuscript.

## Discussion

The main aim of this study was to investigate the relationship between public transport use cessation and two health outcomes – gait speed and cognitive function – in a quasi-causal analysis of longitudinal data. Results from linear mixed effects models suggest that participants who ceased using public transport due to structural reasons experience significantly accelerated health declines in both outcomes analysed.

For gait speed, although statistically significant, the magnitude of the observed effect, -0.001 m/s, seems far from the minimal clinically significant values – of around 0.03 –0.01 m/s [26, 27] – even when accounting for accumulation over years. However, it is important to remember that this effect is an average, and it is likely that certain subgroups within ceasers experience higher magnitudes of effect – specifically, frailer older adults and those who remain without other mobility options after ceasing to use public transport. Moreover, this effect does not replace the natural rate of age-related decline in gait speed, but rather adds to it. This means that in practice, cessation appears to shorten the time it takes for a person to experience meaningful gait speed decline.

For cognitive function, the observed added effect of cessation on cognitive function decline was also small, but more evident. Clear cutoffs for minimal clinically significant changes in cognitive function z-scores were not available in the literature. However, with a conservative assumption of 0.1 standard deviations as the minimally clinically significant value, the added effect becomes significant after approximately 8 years, on average; representing a small, but potentially significant, effect of public transport use cessation on cognitive function.

Previous studies examining the links between public transport use and health in older adults have often been cross-sectional; and even longitudinal studies in this domain often struggled to discern between the two potential causal pathways in these links [17, 19, 28]. One of the main advantages of the current study is the utilisation of the structural ceasers group as an instrumental quasi-experimental subgroup, where cessation is plausibly independent from health. Indeed, our analyses among participants that ceased using public transport due to health-related reasons showed that they experience a significantly different pattern of decline than their structural peers, with decline reported on the same wave as cessation, presumably predating it and being a cause for it, and not vice versa. This can be easily explained by an adverse health event, such as a fall, a stroke, hospitalisation or other events, that simultaneously lead to both gait speed and cognitive declines and public transport use cessation.

Despite its longitudinal character, the current study has some meaningful limitations. First, the measurements available in ELSA bring some weaknesses with them – specifically, gait speed is measured with a 2.4 m trail, rather than longer walks. While short timed walks are highly correlated with longer gait speed tests, it is well established that short walks overestimate gait-speed, and could thus lead to biases in our analyses [29, 30]. While cognitive function is measured in various standardised tests in ELSA, the inconsistency of these tests between waves prevented us from creating a more comprehensive composite score. As a result, the measure used in this study reflects memory and executive function, and might not represent other meaningful dimensions of cognitive function, limiting the clinical relevance of this variable. Second, the exposure variable in this study, public transport use cessation, was constructed solely based upon participants’ self-report; this entails several risks inherent to self-report, including recall bias and other forms of inaccurate reporting. This is also true for the reported reason for cessation – participants could have named structural reasons when in reality other reasons applied, as these external reasons could be perceived as less sensitive or ‘shameful’ than health-related reasons. Additionally, participants might have described their cessation as structural when contextual factors – such as a relocation or passing-away of a partner, for example – led to a change in public transport availability or accessibility, and consequently to cessation. Such contextual factors could independently affect health, confounding the investigated link. Relatedly, a strictly causal interpretation would require structural cessation to be entirely exogenous. Systemic differences in wealth and car access between always users and structural ceasers in our sample hint that structural cessation is at least partly affected by choice, and specifically by transport alternatives. It is possible that under the same exogenous structural pressure, those with alternative mobility options chose to cease using public transport to a larger degree than those without them, reflecting endogenous selection bias, possibly attenuating this study’s conclusions. Third, transport and other social determinants of health are very context dependent [31, 32]. For instance, the average frequency of public transport use varies substantially across contexts, and this frequency may potentially affect the health implications of structural cessation. This study was conducted using data from England, and its conclusions should not be expanded to other context without caution.

Lastly, while this analysis design had the advantage of allowing to carefully draw quasi-causal conclusions regarding the links between cessation and gait speed and cognitive function, it had the disadvantage of working on a relatively small dataset, with the population of structural ceasers being relatively young, healthy, and well-off, socially and economically. This, in addition to the potential selection bias previously mentioned, could have led to an underestimation of the true causal effects of cessation. We hypothesise that the effects observed for public transport use cessation in this study among structural ceasers might be more pronounced among groups with less ‘choice’ – and specifically, those older adults who after ceasing to use public transport lack other means of transportation. The latter presumably experience a higher degree of consequences of the decision to stop using public transport – both physically (such as a decline in physical activity) and socially (such as reduced social participation or independence). ELSA did not contain a sufficient number of participants who stopped using public transport due to structural reasons and who did not have access to a private vehicle. This is a topic for future study, when sufficient data is gathered or when other databases become available.

This study joins a growing body of literature regarding age-friendly transport (e.g., [33, 34]) and public transport use as a social determinant of health in older age [35–37]. While the magnitude of the links identified in this study is relatively small, it is the first to provide initial, directional, quantitative evidence that ceasing to use public transport could lead to adverse health outcomes among older adults. This is important because, at least in some cases, public transport use cessation is not inevitable, and some older adults could continue using it given adequate infrastructure, facilities, and transport options [12, 38]. This study’s findings highlight the importance of answering older adults’ mobility needs, not only for those who cannot drive, but also for those after driving cessation. However, this is only preliminary evidence: future studies should ascertain the magnitude of the links identified in this study, investigate other relevant health outcomes, and examine these findings in other, diverse contexts. Additionally, to facilitate future research, longitudinal databases on ageing should include variables related to transport and cessation, including private and public transport [17, 39].

## Conclusions

Public transport use cessation was found to be linked to a small, but statistically significant, acceleration in the decline of gait speed and cognitive function, in a nationally representative cohort of older adults in England. These findings reinforce recent evidence of the health benefits of public transport use, and underscore the importance of providing adequate, sufficient, and accessible public transport as a public health strategy for the promotion of healthy ageing.

## Data Availability

Data from the English Longitudinal Study of Ageing is available via the UK data service: https://ukdataservice.ac.uk/.

## Abbreviations

ELSA: English Longitudinal Study of Ageing
